# Differential antitumor effects of vitamin D analogues on colorectal carcinoma in culture

**DOI:** 10.3892/ijo.2015.3088

**Published:** 2015-07-17

**Authors:** J.M. WIERZBICKA, A. BINEK, T. AHRENDS, J.D. NOWAC KA, A. SZYDŁOWSKA, Ł. TURCZYK, T. WĄSIEWICZ, P.M. WIERZBICKI, R. SĄDEJ, R.C. TUCKEY, A.T. SLOMINSKI, J. CHYBICKI, K. ADRYCH, Z. KMIEĆ, M.A. ŻMIJEWSKI

**Affiliations:** 1 Department of Histology, Faculty of Medicine, Medical University of Gdańsk, Gdańsk, Poland; 2 Students Scientific Association BIO- MED, Intercollegiate Faculty of Biotechnology of the University of Gdańsk and Medical University of Gdańsk, Gdańsk, Poland; 3 Department of Molecular Enzymology, Intercollegiate Faculty of Biotechnology of the University of Gdańsk and Medical University of Gdańsk, Gdańsk, Poland; 4 School of Chemistry and Biochemistry, The University of Western Australia, Crawley WA, Australia; 5 Department of Dermatology, University of Alabama Birmingham, VA Medical Center, Birmingham, AL 35294, USA; 6 Department of General Surgery, Hospital Ministry Internal Affairs, 80104 Gdańsk, Poland; 7 Department of Hepatology and Gastroenterology, Faculty of Medicine, Medical University of Gdańsk, 80210 Gdańsk, Poland

**Keywords:** vitamin D, calcitriol, vitamin D analogues, colorectal cancer, vitamin D receptor

## Abstract

Colorectal cancer (CRC) is an emerging global problem with the rapid increase in its incidence being associated with an unhealthy lifestyle. Epidemiological studies have shown that decreased levels of vitamin D_3_ significantly increases the risk of CRC. Furthermore, negative effects of vitamin D_3_ deficiency can be compensated by appropriate supplementation. Vitamin D_3_ was shown to inhibit growth and induce differentiation of cancer cells, however, excessive vitamin D_3_ intake leads to hypercalcemia. Thus, development of efficient vitamin D_3_ analogues with limited impact on calcium homeostasis is an important scientific and clinically relevant task. The aims of the present study were to compare the antiproliferative potential of classic vitamin D_3_ metabolites (1α,25(OH)_2_D_3_ and 25(OH)D_3_) with selected low calcemic analogues (calcipotriol and 20(OH)D_3_) on CRC cell lines and to investigate the expression of vitamin D-related genes in CRC cell lines and clinical samples. Vitamin D_3_ analogues exerted anti-proliferative effects on all CRC cell lines tested. Calcipotriol proved to be as potent as 1α,25(OH)_2_D_3_ and had more efficacy than 20-hydroxyvitamin D_3_. In addition, the analogs tested effectively inhibited the formation of colonies in Matrigel. The expression of genes involved in 1α,25(OH)_2_D_3_ signaling and metabolism varied in cell lines analysed, which explains in part their different sensitivities to the various analogues. In CRC biopsies, there was decreased VDR expression in tumor samples in comparison to the surgical margin and healthy colon samples (P<0.01). The present study indicates that vitamin D_3_ analogues which have low calcemic activity, such as calcipotriol or 20(OH)D_3_, are very promising candidates for CRC therapy. Moreover, expression profiling of vitamin D-related genes is likely to be a powerful tool in the planning of anticancer therapy. Decreased levels of VDR and increased CYP24A1 expression in clinical samples underline the importance of deregulation of vitamin D pathways in the development of CRC.

## Introduction

Colorectal cancer (CRC) is the second among women and third among men of the most commonly diagnosed cancers in the world (1). A World Health Organization study from 2012 showed that colorectal cancer exceeds 1,360.500 new cases every year worldwide (1). Furthermore, during the last 5 years, CRC incidence and mortality rates have increased in all developed countries. This appears to coincide with a diet change toward more fat- and carbohydrate-rich foods, and a deficiency in many nutrients including vitamin D. This so-called urban-industrial or Western diet is highly based on processed foods (2).

Current colorectal cancer therapy is based on a combination of cytostatic drugs and surgery where applicable (3). The chemotherapeutic agent of choice is a 5-fluorouracil (5-FU), which is used for the treatment of both the advanced and early stages of colorectal cancer. Unfortunately, the effectiveness of 5-FU monotherapy of colorectal cancer is limited to only 10–15% of cases (4). Identification of compounds that can enhance the response to 5-FU is a key step in the development of combined chemotherapies. Several studies have shown that 1α,25(OH)_2_D_3_ can be administered alone or in combination with other antitumor agents to enhance the efficacy of therapy. For example, 1α,25(OH)_2_D_3_ downregulates the expression of thymidylate synthase (TS) and therefore promotes a cytotoxic response to 5-FU (5). Other studies have demonstrated that 1α,25(OH)_2_D_3_ enhances cellular sensitivity of colorectal cancer cells to 5-FU through a calcium-sensing receptor (5,6).

**Figure 1:**
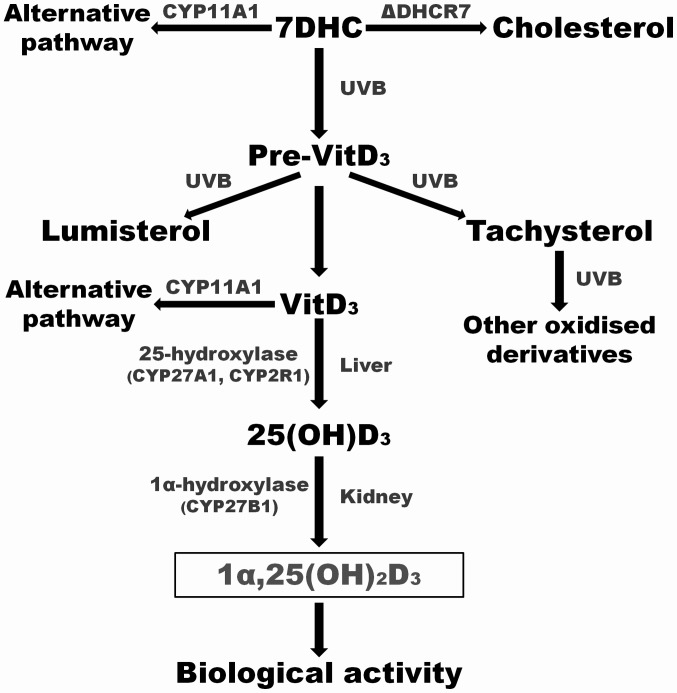
Synthesis, activation and metabolism of vitamin D_3_.

In humans, vitamin D_3_ is formed from 7-dehydrocholesterol (cholesta-5,7-dien-3β-ol, 7DHC) in the basal layer of the epidermis. Initially, the B-ring of 7-DHC undergoes photolysis upon the exposure of skin to UV-B radiation, leading to formation of previtamin D_3_ (7). Pre-vitamin D_3_ then isomerizes to vitamin D_3_, or with further UV-B radiation to tachysterol_3 (_T_3_) and lumisterol_3_ (L_3_). Subsequent activation of vitamin D_3_ involves sequential 25- and 1-α-hydroxylations of vitamin D_3 (_cholecalciferol) which can occur at both the systemic and local levels. In the liver, vitamin D_3_ is hydroxylated by mitochondrial and microsomal 25-hydroxylases (CYP27A1 and CYP2R1) to 25-hydroxyvitamin D_3_ (25(OH)D_3_), also known as 25-hydroxycholecalciferol or calcifediol) (8). 25(OH)D_3_ is then hydroxylated in the kidneys by mitochondrial 1α-hydroxylase (CYP27B1) (9) producing 1α,25(OH)_2_D_3_ (calcitriol), the fully active form of vitamin D_3_ (10). A number of tissues and organs such as intestines and skin can also activate vitamin D_3_ through these two sequential hydroxylations (11). The activity of 25(OH)D_3_ and 1α,25(OH)_2_D_3_ is limited by the 24-hydroxylase (CYP24A1), which initially transforms them to 24,25(OH)_2_D_3_ and 1,24,25(OH)_3_D_3_, respectively, prior to their further oxidation and cleavage of the side chain resultig in complete inactivation (12). Both 24,25(OH)_2_D_3_ and 1,24,25(OH)_3_D_3_ possess lower affinity for the vitamin D receptor (VDR) than 1,25(OH)_2_D_3_ (13). In addition, 7DHC, vitamin D_2_ and vitamin D_3_ can be metabolized by the steroidogenic enzyme, CYP11A1 (14–17). This enzyme catalyses a series of hydroxylations favoring C20, C22 and C23 but only results in cleavage of the side chain in the case 7DHC (18). CYP11A1, in combination with the classic vitamin D hydroxylases (CYP27A1, CYP27B1 and CYP24A1), has recently been shown to generate a series of vitamin D analogs in cells or tissues incubated *ex vivo* with vitamin D precursors (17,19–21), which are biologically active in cell culture (22) and *in vivo (*23) models (19,21,24). Our previous studies showed that the initial and major product, 20(OH)D_3_, is non-calcemic (25,26) with its biologically activity being defined by the nature of cellular target (21–23,25–28). Importantly, 20(OH)D_3_ displays anti-proliferative properties towards melanoma and other types of cancer (26,31). Details of the pathways of vitamin D synthesis and metabolism is presented in Fig. 1.

The active form of vitamin D_3_, 1α,25(OH)_2_D_3_, plays a crucial role in calcium homeostasis, ensuring proper functioning of bones, muscles and the nervous system. In the colon it increases the absorption of calcium, in bones it stimulates release of calcium and phosphate, while in kidneys together with parathyroid hormone it regulates calcium reabsorption (32). In addition, 1α,25(OH)_2_D_3_ is involved in regulating the immune system including reducing the inflammatory response (33) and influencing the growth and differentiation of mononuclear cells (34,35). Several vitamin D analogs are already used for the treatment and/or prevention of various diseases such as rickets, osteoporosis and psoriasis (36). The importance of vitamin D is emphasized by a growing body of evidence that there is low 25(OH)D_3_ in the serum of patients with many types of cancer. Therefore, secosteroids (vitamin D and its derivatives), have potential applications in the treatment and/or prevention of several types of cancer, including breast, prostate, colorectal cancers and melanoma (37–39).

Active metabolites of 1α,25(OH)_2_D_3_ are characterized by broad and diverse biological activities. After entering the cell, 1α,25(OH)_2_D_3_ binds to the vitamin D receptor (VDR) which heterodimerizes with the 9-*cis*-retinoic acid receptor (retinoid X receptor, RXR). The VDR-RXR complex acts as a transcription factor, which recognizes a consensus DNA sequence, the vitamin D responsive element (VDRE), and regulates the expression of over 200 genes (13,40). A wide-range of studies show that 1α,25(OH)_2_D_3_ regulates a number of genes involved in cell proliferation, DNA repair, differentiation, apoptosis and membrane transport. Among the most important ones are genes associated with vitamin D_3_ metabolism, as well as with calcium and phosphorus homeostasis (41). One of the most well-described activities of 1α,25(OH)_2_D_3_ is the stimulation of the expression of the *CYP24A1* gene (coding 24-hydroxylase) and the inhibition of the expression of the *CYP27A1* (25-hydroxylase) and *CYP27B1* (1α-hydroxylase) genes (42). It is therefore likely that variations in the expression of genes involved in the regulation of 1α,25(OH)_2_D_3_ activity could play an important role in determining susceptibility to CRC.

It has been suggested that 1α,25(OH)_2_D_3_ can act via an alternative signaling pathway which involves binding to the endoplasmic reticulum protein PDIA3 (also known as ERp57) (43). PDIA3 protein belongs to the protein disulfide isomerases family (PDI) and is a disulfide oxidoreductase. PDIA3 is involved in the transport and post-translational modification of glycoproteins, in particular the heavy chains of the major histocompatibility complex type I (MHC I) and tyrosinase (44). In addition, calcium and potassium absorption in the colon is stimulated by 1α,25(OH)_2_D_3_ via PDIA_3_. It is also interesting that the PDIA3 contains a nuclear localization signal sequence and a DNA binding domain, and interacts with the transcription factor, STAT3 (45). It has also been shown that under specific conditions PDIA3 can acts as a transcription factor or co-activator.

A number of studies support the potential preventative and therapeutic effects of vitamin D_3_, the precursor to 1α,25(OH)_2_D_3_, for anticancer treatment. Unfortunately, administration of vitamin D_3_ at its effective therapeutic dose during treatment (>50,000 units/day) is associated with a high probability of hypercalcaemia occurrence. This can cause such side-effects as nausea, vomiting and loss of appetite (46,47), or be lethal depending on the anatomical site of calcium deposition. For this reason many laboratories have conducted studies on 1α,25(OH)_2_D_3_ analogues with little or no impact on the calcium homeostasis, but still retaining therapeutically crucial properties (37). In the present study we have examined the effects of 1α,25(OH)_2_D_3_, and selected analogues with low calcemic activity [calcipotriol, 25(OH)D_3_, 20(OH)D_3_], on CRC cell lines (LoVo, HT29 and HCT116). We also examined the expression of key genes associated with 1α,25(OH)_2_D_3_ activity and metabolism in CRC cell lines and colon cancer biopsies.

## Materials and methods

### Cell culture

The CRC cell lines LoVo (colon cancer), HT29 (colorectal adenocarcinoma) and HCT116 (colorectal carcinoma) were purchased from ATTC (Wesel, Germany). The HT29 cell lines were cultured in McCoy's medium, LoVo were cultured in Dulbecco's modified Eagle's medium (DMEM) and F-10 medium (ratio 1:1), while HCT116 were grown in DMEM only. All media were supplemented with 10% fetal bovine serum (FBS; Sigma) and 1% PSA (penicillin-streptomycin-amphotericin B solution; Sigma). Cell cultures were maintained in a 37°C humidified atmosphere of 5% CO_2_. Cells were passaged every 2–3 days.

### Patients

The study was approved by the local ethics committee, and informed, written consent regarding the use of tissue was obtained before surgery or colonoscopy from all CRC patients and control individuals, respectively (48). The CRC specimens were received from the Department of General Surgery, Hospital Ministry Internal Affairs in Gdansk whereas the control samples were collected from the Department of Hepatology and Gastroenterology of the Medical University of Gdansk (MUG). Clinical and demographical data were collected at the time of enrollment. The study included 20 patients with CRC (11 males and 9 females; mean age 67.2±10.8 years; range, 50–87 years). None of the CRC patients had a second neoplastic disease or had a history of previous chemo- or radiotherapy. Tumor CRC samples were collected from: rectum (n=7 cases), sigmoid colon (n=5), transverse (n=5) and 3 cases from cecum/ascending colon. The control group comprised 4 healthy individuals (1 males and 3 females; mean age 59±7.9 years; range, 54–65 years) who underwent colonoscopy as a part of routine surveillance for CRC. None of the CRC patients or controls suffered from inflammatory bowel disease or had a family history of CRC. Patients were not on medications at the time of the investigation.

### Vitamin D and its analogues

**Figure 2:**
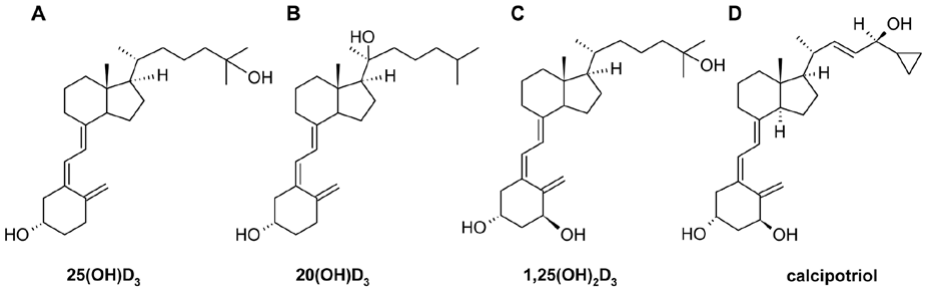
Structures of the vitamin D analogues used in the present study. (A) Calcifediol (25(OH)D_3_), (B) 20(OH)D_3_, (C) calcitriol (1,25(OH)_2_D_3_), (D) calcipotriol.

Calcitriol (1α,25(OH)_2_D_3_) and calcifediol (25(OH)D_3_) were obtained from Sigma (Poznań Poland). Calcipotriol was obtained from Pharmaceutical Research Institute (Warsaw, Poland). 20*S*-Hydroxyvitamin D_3_ (20(OH)D_3_; (3β,5*Z*,7*E*)-9,10-secocholesta-5,7,10(19)-trien-3,20-diol) was synthesized and purified as previously described (15). The structures of these secosteroids are presented in Fig. 2. They were dissolved in 99.5% ethanol and stored at −20°C. Their concentration was calculated using an extinction coefficient of 18,200 M^−1^cm^−1^ at 265 nm. For *in vitro* use, dilutions were made in the same medium as those for cell culture. The highest final concentration of ethanol used in the present study never exceeded 0.2% and had no effect on cell growth.

### Proliferation assays

The degree of proliferation of cells following treatment was measured using the 3-(4,5-dimethylthiazol-2-yl)-2,5-diphenyltetrazolium bromide assay (MTT) and the sulforhodamine B assay (SRB). Cells were seeded at a density of ~7×10^3^ (LoVo and HCT116) or 10^4^ (HT29) cells per 100 μl/well in 96-well plates in the appropriate growth medium, supplemented with 1% PSA and 2% charcoal-treated FBS, and allowed to attach for 24 h before treatment. The medium was replaced with fresh medium containing serial dilutions of 1α,25(OH)_2_D_3_ or its analogues (0.01 nM - 1 μM) in a volume of 100 μl/well. The plates were incubated at 37°C for an additional 48 h. For the MTT assay, 20 μl of MTT (5 mg/ml) were added to each well. After incubation for 4 h, the medium was removed and 100 μl of the solubilisation solution (1 M acidic isopropanol) was added to each well. After incubation for 5 min the absorbance was measured at 570 nm using a microplate reader. For the SRB assay, after incubation with the vitamin D analogs, 100 μl of 10% trichloroacetic acid (TCA) was added to each well and plates were incubated for 1 h at 4°C. Afterwards, the medium was removed and cells were washed 5 times with deionized water. Following overnight air-drying, 100 μl of SRB solution [0.4% (w/v) in 1% acetic acid] was added to each well. After incubation for 15 min plates were washed 5 times with 1% acetic acid and air-dried. The protein-bound dye was solubilised with a 10 mM Tris-base solution (pH 10.5). The absorbance of the dye was recorded at 570 nm with a microplate reader.

### Cell growth in 3D matrigel

The three-dimensional cell growth assay was performed in a Matrigel Matrix (BD Bioscience, Heidelberg, Germany). Two drops of cell suspension in Matrigel (~2 mg of protein/ml, ~1.5×10^3^ cells/40 μl) were added per well to a 12-well tissue culture plate and plates incubated at 37°C for up to 30 min to allow the Matrigel to solidify. Wells were then submerged in growth medium supplemented with the relevant concentrations of 1α,25(OH)_2_D_3_ or it analogs (final concentrations: 0.5, 0.1 or 0.2 μM). Growth medium was replaced every third day. Following 10 days of incubation representative pictures were taken and relative colony size was determined for 50 random colonies using ImageJ software. Each experiment was repeated at least four times. Due to the fast growth rate and the formation of crypt-like structures, only the inhibition of the formation of ‘megacolonies' was compared.

### Sample collection

CRC and control samples were collected as previously described with some modifications (48). Briefly, CRC and surgical margin tissue samples from each CRC patient collected during surgery were placed in sterile vials containing 5 volumes of RNAlater buffer (Ambion-Life Technologies, Grand Island, NY, USA), incubated for 6 h at 4°C and then stored at −25°C until further analysis. The same procedure was also applied to control colon biopsies.

### cDNA preparation and PCR assays

RNA was extracted using a Total RNA Mini Plus kit (A&A Biotechnology, Gdynia, Poland) according to the manufacturer's protocol. Before the RNA isolation, tissue samples were homogenized in 2 ml vials with the use of ceramic beads (Blirt-DNA Gdansk, Gdansk, Poland) in the MagNA Lyser apparatus (Roche Diagnostics Deutschland GmbH, Mannheim, Germany) for 45 sec at 6,000 rpm. The concentration and purity of isolated RNA was measured using a NanoDrop^®^ spectrophotometer. A total of 2 μg of RNA were subjected to reverse transcription using a RevertAid™ First Strand cDNA Synthesis kit (Thermo Fisher Scientific Inc., Waltham, MA, USA). All the primer sequences (Table I) were designed by the authors using Primer BLAST database and were purchased from Sigma-Aldrich (Munich, Germany). Quantification of the expression of genes of interest was carried out using the StepOnePlus™ Real-Time PCR System (Life Technologies-Applied Biosystems, Grand Island, NY, USA) with SYBR^®^ Green as a fluorophore. qPCR reactions were performed in a total volume of 20 μl using Real-Time HS 2× PCR Master Mix SYBR^®^ kit (A&A Biotechnology), 2 μl of cDNA diluted 4-fold and 200 nM of each primer pair. The PCR conditions were: 95°C for 5 min followed by 40 cycles of denaturation for 15 sec at 95°C, annealing for 20 sec at 59°C, extension for 15–25 sec at 72°C, and fluorescence reading for 5 sec at 77°C. Dynamic melting curve analysis was performed for all reactions. All qPCR reactions were performed in duplicate and data were collected with a StepOnePlus™ software version 2.2 (Life Technologies). The expression of the genes was normalized by comparative ΔΔ-C_t_ method, using β-actin as a housekeeping gene, followed by calibration (fold-change) to normalized expression data of samples from controls (ratio=1).

### Western blotting

Cells were scraped and lysed in the presence of ice-cold Laemmli buffer supplemented with protease inhibitor cocktail. Protein concentrations were determined by the Bradford assay. An equal amount of protein from each sample (50 μg) was loaded per lane, proteins were separated by SDS-PAGE and then transferred onto an Immun-Blot™ PVDF membrane (Bio-Rad Laboratories, Hercules, CA, USA). The membranes were incubated with primary antibodies: anti-VDR (polyclonal, 1:1,000; Sigma-Aldrich, SAB2102673), anti-CYP24A1 (polyclonal, 1:500; Santa Cruz Biotechnology, sc-66851) or HRP conjugated anti-β-actin antibody (monoclonal, 1:5,000; Santa Cruz Biotechnology, sc-47778) overnight at 4°C. After three washes in TBST, secondary mouse anti-rabbit antibodies conjugated to horseradish peroxidase (1:10,000; Santa Cruz Biotechnology, sc-2537) were added and following incubation for 1 h at room temperature. Blots were developed with SuperSignal^®^ West Pico chemiluminescent substrate (Thermo Fisher Scientific) according to the manufacturer's protocol.

### Statistical analysis

Data were analyzed with the Student's t-test (for two groups) or one-way analysis of variance and appropriate post hoc test (the ANOVA Kruskal-Wallis test for comparison of several groups), using Statistica (StatSoft Inc., Tulsa, OK, USA) or GraphPad Prism v6.03 (GraphPad Software, San Diego, CA, USA). For tissue samples, the Mann-Whitney U test of Kruskal-Wallis ANOVA was applied since the qPCR results for those specimens did not pass the omnibus test of D'Agostino and Pearson (49). Such procedures allowed the comparison of each experimental group with the control. Spearman's test was applied to check the association of paired data (correlation test). The data are presented as mean ± SD or median value with range (min-max). Results were considered statistically significant at P<0.05 and are marked in the figures as: ^****^P<0.0001; ^***^P<0.001; ^**^P<0.01; ^*^P<0.05.

## Results

### The effect of 1α,25(OH)_2_D_3_ and its analogs on the proliferation of CRC cell lines

**Figure 3:**
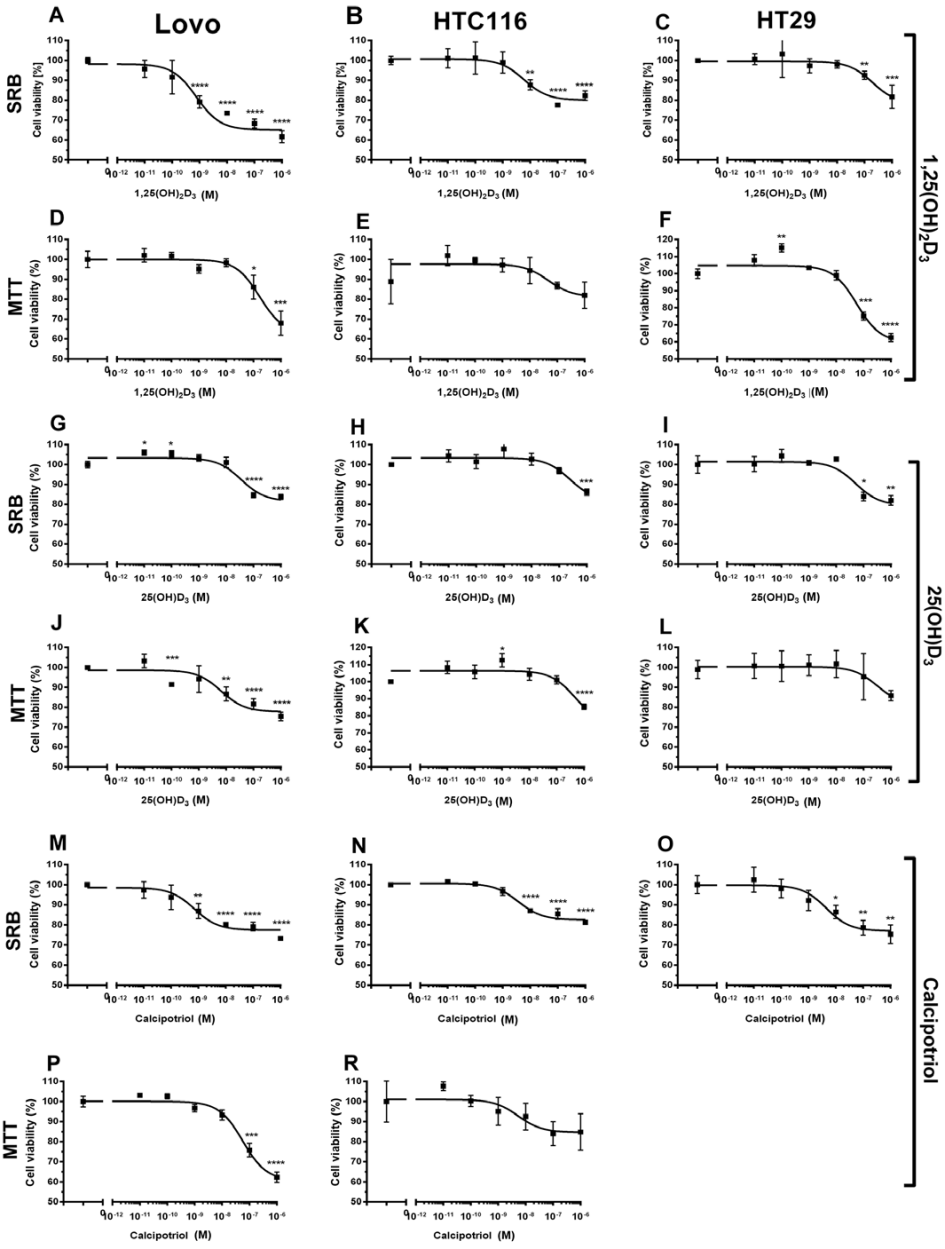
The effect of 1,25(OH)_2_D_3_, 25(OH)D_3_ and calcipotriol on the proliferation of CRC cell lines. The effects of (A–F) 1,25(OH)_2_D_3_, (G–L) 25(OH)D_3_ and (M–R) calcipotriol on proliferation of LoVo, HCT116 and HT29 cells were measured by MTT and SRB assays. Cells were incubated in medium (see Materials and methods) with varying concentrations (10^−6^ to 10^−11^ M) of the vitamin D analogue for 48 h prior to the SRB and MTT assays. Significance compared to the vehicle-treated control: ^*^P<0.05, ^**^P<0.01, ^***^P<0.001, ^****^P<0.0001; mean ± SD.

The effects of vitamin D analogues on the inhibition of the proliferation of CRC cell lines, LoVo, HT29 and HCT116, were assessed by means of MTT and SRB assays (Fig. 3 and Table I). As shown by the MTT assay, the inhibitory effect of 1α,25(OH)_2_D_3_ treatment was similar for LoVo and HT29 cell lines (Fig. 3A and C). A significant decrease in cell growth compared to vehicle-treated cells (P<0.001 for LoVo and P<0.0001 for HT29 cell line) was seen with 1 μM 1α,25(OH)_2_D_3_ (Fig. 3D and F), but not for HCT116 cells where the inhibition of growth did not reach statistical significance (Fig. 3E). The IC_50_ value of 1,25(OH)_2_D_3_ for LoVo (168 nM) with the MTT assay was slightly higher than that for HT29 cells (57 nM) and HCT116 cells (47 nM) (Table II).

The IC_50_ values measured for 1,25(OH)_2_D_3_ by the SRB assay differed to those measured using the MTT assay, presumably due to the different biochemical parameters measured by these assays, the activity of NAD(P)H-dependent cellular oxidoreductase enzymes in the case of MTT and cellular protein content in the case of SRB. The proliferation of the LoVo cell line (Fig. 3A) measured with the SRB assay was significantly (P<0.0001) decreased with 1.0 nM 1,25(OH)_2_D_3_ compared to vehicle-treated control cells, with the calculated IC_50_ being 0.77 nM (Table III). A statistically significant decrease in the proliferation of HCT116 and HT29 cells by 1,25(OH)_2_D_3_ was also seen using the SRB assay (Fig. 3B and C) although IC_50_ values were considerably higher than for the LoVo cells (5.9 and 196 nM, respectively (Table II).

Calcipotriol, a low-calcemic analogue of vitamin D (39), proved to be the most potent inhibitor of proliferation of the three analogs tested using both the MTT and SRB assays on the colorectal cancer cell lines (Fig. 3M–R and Table II). With the SRB assay, calcipotriol caused significant inhibition of the proliferation of the LoVo, HCT116 and HT29 cells, compared to vehicle-treated cells, at concentrations as low as 1, 10 and 10 nM, respectively (Fig. 3M–O).

The potency of 25(OH)D_3_ relative to 1,25(OH)_2_D_3_ varied for the LoVo and HCT116 cells depending on whether the MTT or SRB assay was used (Fig. 3). It should be noted that colon cells express CYP27B1 and are capable of transforming 25(OH)D_3_ to 1,25(OH)_2_D_3_ (20). With both the MTT and SRB assays, 25(OH)D_3_ displayed a lower potency than 1,25(OH)_2_D_3_ for inhibiting the proliferation of HCT116 cells (Table II), likely reflecting the relatively poor ability of these cells to activate this secosteroid by 1α-hydroxylation (Table II).

### Effect of 1α,25(OH)_2_D_3_ and its analogues on CRC cell lines colony formation in three-dimensional Matrigel

The LoVo and HT29 cell lines were used to test the effects of 1α,25(OH)_2_D_3_ and its two low- or non-calcemic analogues, calcipotriol and 20(OH)D_3_, respectively, on cell growth and colony formation in three-dimensional Matrigel. Our initial tests with the HCT116 cell line showed that these cells grew too slowly in Matrigel to perform comparable experiments so this particular cell line was excluded from the Matrigel assays. In control experiments (without treatment), the majority of the LoVo and HT29 cells gave rise to large colonies with complex structure ‘megacolonies', while the remaining ~10% of cells formed small colonies with limited growth. Due to the faster growth rate with the formation of crypt-like structures, only the inhibition of the formation of ‘megacolonies' was analyzed.

**Figure 4:**
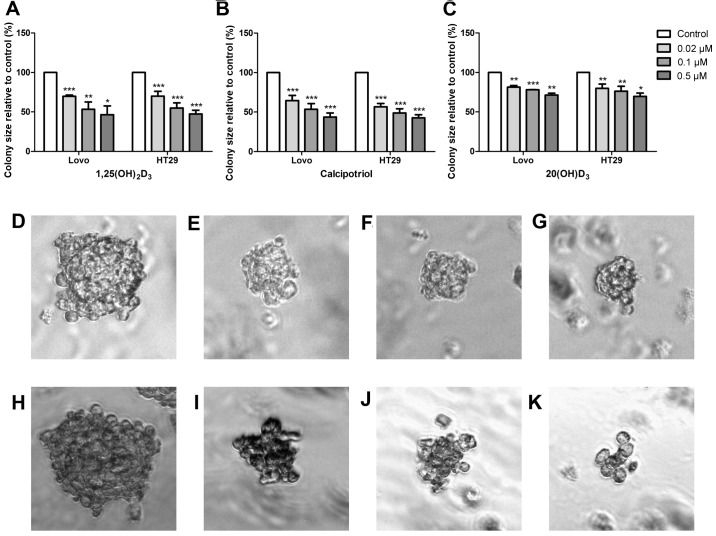
The effect of 1α,25(OH)_2_D_3_, calcipotriol and 20(OH)D_3_ on CRC colony formation in Matrigel. Cells in Matrigel were incubated with varying concentrations (0.02, 0.1 or 0.5 μM) of (A) 1α,25(OH)_2_D_3_, (B) calcipotriol or (C) 20(OH)D_3_ for 10 days. Light microscopy of representative ‘megacolonies' formed in 10 days following the addition of (D–G) 1α,25(OH)_2_D_3_ and (H–K) calcipotriol on LoVo cells are also shown. Original magnification, ×100. The concentrations of 1α,25(OH)_2_D_3_ and 25(OH)D_3_ used were 0 μM (controls, D and H); 0.02 μM (E and I); 0.1 μM (F and J) and 0.5 μM (G and K). Significance compared to the control: ^*^P<0.05, ^**^P<0.01, ^***^P<0.001.

In agreement with the results of the MTT and SRB assays, vitamin D analogues significantly diminished cell proliferation as shown by the decrease in both size and the number of the colonies formed in a presence of the three analogues tested (Fig. 4). Over the limited range of concentrations tested (20–500 nM), the potency of 1,25(OH)_2_D_3_, calcipotriol and 20(OH)D_3_ were reasonably similar with a significant reduction in colony size seen in both LoVo and HT29 cells for all three analogs at a concentration of 20 nM. The magnitude of the inhibition (efficacy) varied between the three analogs. Calcipotriol caused the greatest inhibition (~57%) at the highest concentration tested (500 nM), while 1,25(OH)_2_D_3_ caused ~53% inhibition and 20(OH)D_3_ ~28% inhibition at this concentration, for both cell lines.

### 1,25(OH)_2_D_3_ modulates the expression of some of the genes involved in vitamin D metabolism and signalling

**Figure 5:**
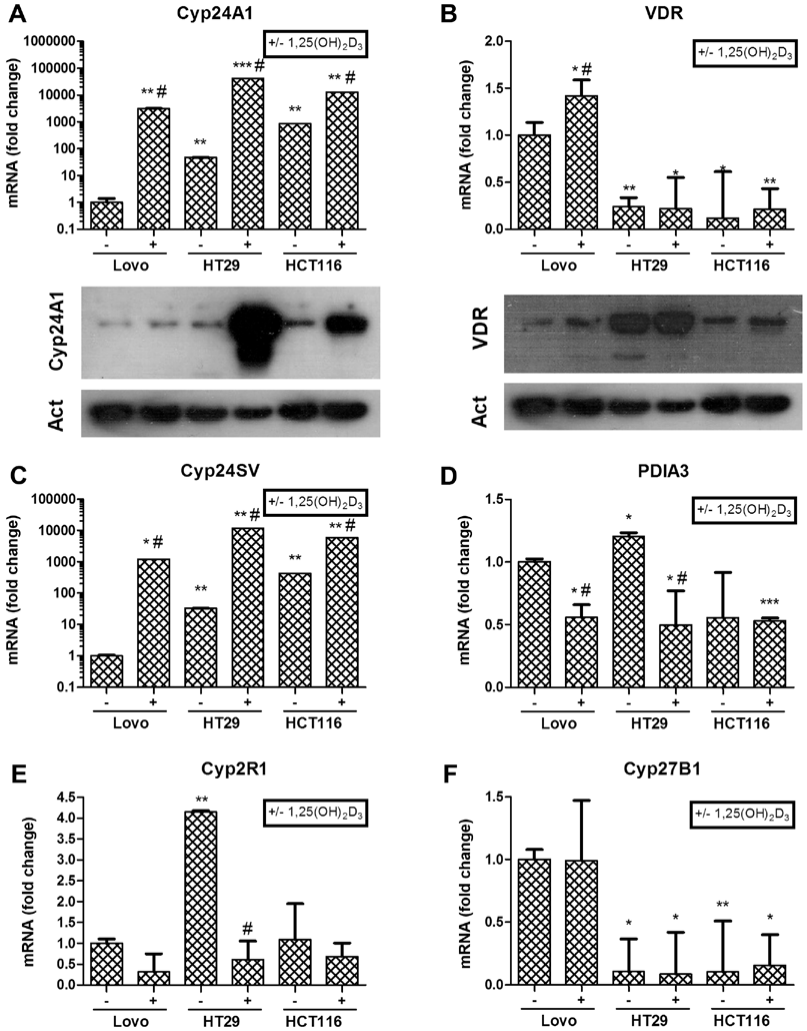
The effects of 1,25(OH)_2_D_3_ on the expression of genes involved in vitamin D_3_ signaling and metabolism. CRC cell lines were incubated with 0.1 μM 1,25(OH)_2_D_3_ for 24 h and the expression of 24-hydroxylase (*CYP24A1* gene) (A), vitamin D receptor (*VDR* gene) (B), splicing variant of 24-hydroxylase (*CYP24SV* gene) (C), protein disulfide isomerase (*PDIA3* gene) (D), 25-hydroxylase (*CYP2R1* gene) (E) and 1α-hydroxylase (*CYP27B1* gene) (F) was analysed by real-time PCR (qPCR). Data were normalized relative to β-actin mRNA and further normalized against the basal expression in LoVo cells. CYP24A1 (A, lower panel) and vitamin D receptor (B, lower panel) proteins were measured by western blotting, with β-actin used as a control. The data are presented are mean ± SEM. Significance level relative to the LoVo cell basal expression: ^*^P<0.05, ^**^P<0.01, ^***^P<0.001. Significance level relative to the untreated control for each cell line is indicated as ^#^P<0.05.

The expression levels of several genes encoding proteins inolved in vitamin D signalling (VDR and PDIA3) and metabolism (CYP24A1 and its splice variant, CYP24SV; CYP27B1 and CYP2R1) were compared by real-time PCR (qPCR) (Fig. 5). Basal levels of transcripts of CYP24A1 and its splice variant, CYP24SV, were strongly elevated in the HT29 and HCT116 cell lines compared to LoVo cells (Fig. 5A and C). 1,25(OH)_2_D_3_ caused a marked and statistically significant elevation of transcript levels for both CYP24A1 and CYP24SV, most pronounced in the LoVo cell line (3,100-fold for CYP24A1 and 1,200-fold for CYP24SV) due to the low basal level of expression. The stimulation of CYP24A1 expression by 1,25(OH)_2_D_3_ was confirmed at the protein level for all three cell lines (Fig. 3A, bottom panels) although the magnitude of the stimulation appeared less for the LoVo cells than at the transcript level.

For the three cell lines, expression of CYP2R1 transcripts was significantly downregulated by 1,25(OH)_2_D_3_ only in the HT29 cells (6.6-fold, P<0.05) where the basal level was higher than in the other cells (Fig. 5E). 1,25(OH)_2_D_3_ did not alter the level of transcripts for CYP27B1 in any of the three cell lines (Fig. 5F). Basal levels of CYP27B1 transcripts were significantly lower in HT29 and HCT116 cells than in LoVo cells. For PDIA3 transcript, basal levels were significantly higher in HT29 cells than in LoVo cells (Fig. 5D). 1,25(OH)_2_D_3_ significantly decreased the the expression of PDIA3 in LoVo and HT29 cells, but not in the HCT116 cell line. 1,25(OH)_2_D_3_ significantly increased the expression of the VDR at the mRNA level in only the LoVo cells. Basal expression was also significantly higher in LoVo cells than in the HT29 or HCT116 cells (Fig. 5B). Consistent with the results at the transcript level, marginal increases at best were seen for VDR expression at the protein level following treatment with 1,25(OH)_2_D_3_. The highest expression of VDR was in the HT29 cells (Fig 3B, lower panels). The splice variant of the VDR protein (lower band) was observed most prominently in untreated HT29 cells.

### Expression of VDR and CYP24A1 genes in CRC biopsies

**Figure 6:**
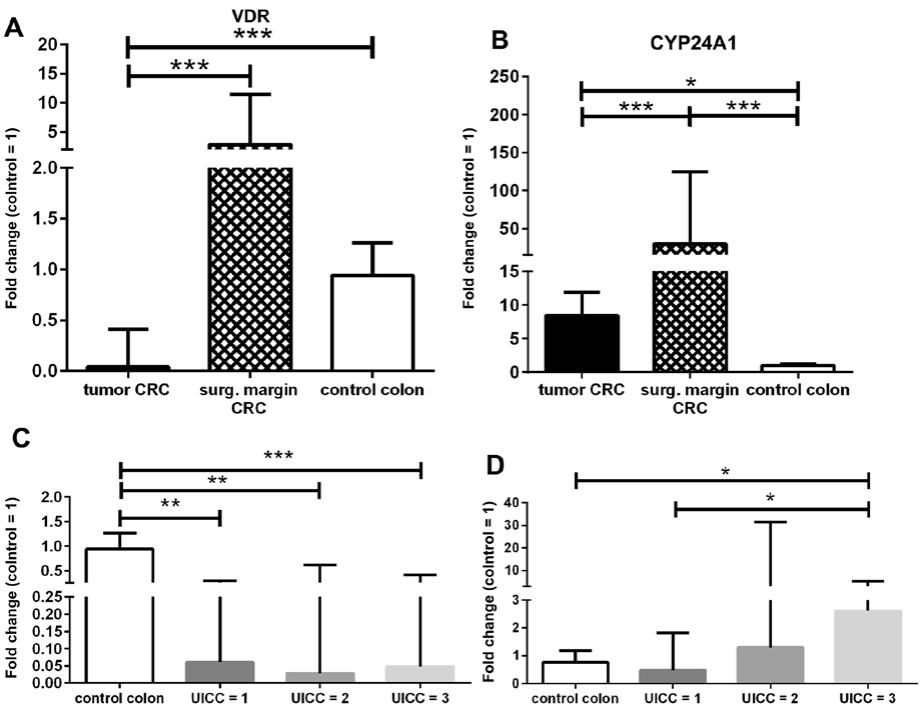
Analysis of VDR and CYP24A1 expression at the mRNA level in clinical samples. Boxes represent median values; (A) VDR and (B) CYP24A1 levels in tumor CRC (black bars) and surgical margin samples (chequered bars) from 20 CRC patients were compared to colon samples from control patients (white bars, set to 1). CRC samples were divided by UICC grades (Union for International Cancer Control) for comparison to control samples for (C) VDR and (D) CYP24A1. Mann-Whitney U tests were applied; significance level, indicated by horizontal lines: ^*^P<0.05, ^**^P<0.01, ^***^P<0.001.

The expression of VDR and *CYP24A1* genes in CRC biopsies and control samples were assessed by qPCR (Fig. 6). Matched tumor-surgical margin biopsies from 20 CRC patients were analyzed in comparison to healthy mucosal colon biopsies from 4 individuals who underwent routine colonoscopy. There was a significant decrease in the expression of VDR in tumor CRC samples in comparison to the surgical margin and healthy control specimens (Fig. 6A and C). Analysis of the clinical data revealed that there was no association between the VDR expression level and either gender, age, tumor progression (Fig. 6C), G staging or metastasis presence.

The highest expression of CYP24A1 was observed in surgical margin specimens in comparison to both tumor and control samples (P<0.0001; Fig. 6B). No correlation between CYP24A1 expression and gender or age was found. When the clinical data were taken into consideration, we observed that CYP24A1 expression was associated with tumor location (proximal to distal colon) (rs=0.48; p<0.02). Despite the general decrease in CYP24A1 mRNA in tumor specimens compared to the surgical margin, we found increased expression in higher (Stage I to III, UICC grading 1 to 3) developed tumors (rs=0.36; P<0.05, Spearman's test, P=0.001; Kruskal-Wallis ANOVA; Fig. 6D). Finally, CYP24A1 expression in tumor tissue was correlated with VDR mRNA levels (rs=0.57, P<0.05; Spearman's test; Fig. 6C and D).

## Discussion

The first aim of the study was to investigate the effects of 1,25(OH)_2_D_3_ and its low-calcemic analogues (particularly calcipotriol and 20(OH)D_3_) on the proliferation of CRC cell lines. Calcipotriol has a 100- to 200-fold lower effect on calcium metabolism than 1,25(OH)_2_D_3_ (51), while 20(OH)D_3_ is non-calcemic at concentration ≤60 μg/kg (26). Three different CRC cell lines were chosen, representing colon cancer (LoVo), colorectal adenocarcinoma (HT29) and colorectal carcinoma (HCT116). The LoVo cell line is well differentiated, HT29 cells are moderately differentiated, while the HCT116 cell line is poorly differentiated (52).

Overall, our results show that calcipotriol has similar or greater potency to 1,25(OH)_2_D_3_ using MTT, SRB and Matrigel assays of cell proliferation. 25(OH)D_3_ was generally found to be less potent than 1,25(OH)_2_D_3_ but there were exceptions such as in the MTT assay on LoVo cells and the SRB assay on HT29 cells. It should be noted that while it is generally accepted that 25(OH)D_3_ exerts its action following its conversion to 1,25(OH)_2_D_3_ by CYP27B1, which is present in the colon, recent studies indicate that it may have distinct actions independent of 1,25(OH)_2_D_3_ (53).

The treatment of the LoVo and HT29 cell lines with 1,25(OH)_2_D_3_, calcipotriol or 20(OH)D_3_ in Matrigel caused a statistically significant inhibition of colony size at a concentration of only 20 nM, with stronger inhibition at 500 nM. Since matrigel resembles a three dimensional basement membrane complex into which cells can invade, proliferate and grow (54), these results indicate that the low calcemic analogs of vitamin D_3_ tested in the present study are worthy of future *in vivo* testing.

The SRB assay results indicate that the LoVo cell line is the most sensitive to the vitamin D analogues examined (lowest IC_50_ values), while the HTC116 cell line was the most resistant. Thus, it might be speculated that moderately differentiated cells (LoVo and HT29 cell lines) are more sensitive to vitamin D analogs compared with poorly differentiated HCT116 cells. However, this correlation does not hold for the MTT assay results.

The dose-dependent, anti-proliferative activity of 1α,25(OH)_2_D_3_ on human CRC cells has been previously reported by other researchers (35,55–59). For instance, treatment of the colon cancer cell line, Caco-2, with vitamin D_3_ analogs resulted in growth inhibition by 20–40% (at a concentration of 0.01 μM) (60). This is in general agreement with our results with calcipotriol and 1,25(OH)_2_D_3_, but we observed that IC_50_ values varied between the different cell lines studied. Thus, the observed differences in sensitivity of CRC to vitamin D analogues may dependent on the unique properties of the cancer cells (37,38), including the degree of expression of VDR and CYP24A1 (49), as well as on the molecular structure of the vitamin D_3_ analogue. It has recently been suggested that epigenetic changes may influence the expression of crucial genes which encode proteins involved in the metabolism of vitamin D, such as CYP24A1 (61). According to the classical pathway, vitamin D_3_ acts through the VDR, as supplementation of VDR knockout animals with vitamin D_3_ analogues does not protect them from tumor development (62). Our results indicate that the CRC cell lines studied differ in their constitutive level of VDR mRNA and protein. The LoVo cell line showed the highest relative level of VDR mRNA whereas the HT29 cell line showed the highest concentration of protein. Thus, the relatively high level of VDR mRNA in the LoVo cell line did not correlate with the highest level of its protein product. In fact, the LoVo cell line showed the lowest concentration of VDR protein in comparison to HT29 and HTC116 cells. These inconsistencies can be explained by the recent report showing that the VDR can be regulated at the post-translational level via proteosomal degradation in a cell-type specific manner (63).

Notably, epidemiological studies show that vitamin D deficiency (64), as well as specific polymorphisms in the VDR gene (65,66), are strong prognostic factors for development and severity of CRC. It is also well established that the VDR is expressed by normal colon epithelial cells, but its expression is decreased during the progression of colon cancer (67). A similar reverse correlation between VDR expression and the aggressiveness of the tumor was reported for human melanomas (68,69). Our results reveal that there is a significant downregulation of VDR expression in patients with CRC at stages I to III. Another interesting observation is the comparatively high level of VDR transcript expression in surgical margin specimens in comparison to both tumor and control samples of healthy colon mucosa. A correlation between tumor progression and CYP24A1 expression at the mRNA level was observed in the CRC biopsies. Our results therefore indicate that changes in expression of the above genes may provide useful information as a prognostic predictor for CRC metastasis.

The responsiveness of the CRC cell lines to vitamin D_3_ analogues may also be affected by their metabolism and inactivation. Our *in vitro* results show that there is correlation between the expression of VDR, and to a lesser degree CYP24A1, at the mRNA and protein levels in CRC cell lines treated with 1,25(OH)_2_D_3_. This is in an agreement with the data of Höbaus *et al* (61) who showed a very high level of expression of CYP24A1 in HT29 cells and suggested that this expression is connected with methyltransferase and some histone deacetylase inhibitors in a cell line-dependent manner. Furthermore, the present study shows that basal expression of CYP24A1 and CYP24SV (isoform lacking mitochondrial translocation signal (50) are highly upregulated at the mRNA and protein levels in HT29 and HCT116 cell lines compared to the LoVo cell line. Increased expression of CYP24A1 in cancer cells may attenuate the effects of 1α,25(OH)_2_D_3_ on growth inhibition and differentiation. Stimulation by 1α,25(OH)_2_D_3_ significantly increased the expression of CYP24A1 in LoVo, HT29 and HCT116 cell lines. It has been shown before that induction of CYP24A1 expression by 1α,25(OH)_2_D_3_ treatment is more pronounced in responsive CRCs and melanomas in comparison to resistant ones (56,70,71). Furthermore, CRC cell lines (HT29 and HCT116) with the highest basal expression of CYP24A1 at the transcript level were shown to be less responsive to vitamin D treatment, which is consistent with other studies (35). However, this correlation is not so clear at the protein level. The role of CYP24A1 in tumor biology may be complex as indicated by clinicopathological studies on patients with melanoma (72).

Although, catabolism of vitamin D by CYP24A1 is considered as a major regulatory mechanism controlling 25(OH)D_3_ and 1,25(OH)_2_D_3_ concentrations, expression of other genes involved in vitamin D metabolism may influence the responsiveness of cancer cells to the treatment (57). Recently, it was shown that CYP2R1 and not CYP27A1 is the major vitamin D 25-hydroxylase (73). We have found that that the treatment of HT29 cells with 1,25(OH)_2_D_3_ significantly decreased the expression of CYP2R1 at the mRNA level but the small decreases in the other cell lines did not reach statistical significance. Notably, in the responsive HT29 cells, the basal level of CYP2R1 mRNA was 4 times higher than in the HTC116 or LoVo cells.

1,25(OH)_2_D_3_ had no effect on the expression of 1α-hydroxylase (CYP27B1) at the mRNA level in any of the three cell lines analysed. It is well established now that CYP27B1 is expressed at extra-renal sites such as normal colon, brain, placenta, pancreas, lymph nodes and skin (74). Our data show that basal expression of CYP27B1 at the mRNA level is higher in LoVo cells compared to the HT29 and HCT116 cell lines. Higher CYP27B1 expression has previously been reported in well-to-moderately differentiated states of colon cancer compared to poorly differentiated colon carcinomas (75–77). A similar reverse correlation between CYP27B1 expression and tumor progression and aggressiveness was found in melanomas and ovarian cancer (78,79). Increased expression of CYP27B1 in cancer tissues may convey some chemoprevention to these cancers due to increased conversion of 25(OH)D_3_ of 1,25(OH)_2_D_3_. Notably, the LoVo cells which show the highest expression of CYP27B1, display the lowest IC_50_ of the three cell lines for the inhibition of proliferation by 25(OH)D_3_.

The VDR is not the only target for active forms of vitamin D in the cells. For instance, vitamin D_3_ may exert some non-genomic actions mediated by PDIA3 (80). The most resistant cell line, HCT116, showed the lowest basal level of PDIA3 mRNA and the treatment with 1,25(OH)_3_D_3_ did not affect its expression. In contrast, treatment of LoVo and HT29 cells with 1,25(OH)_2_D_3_ resulted in a significant decrease of PDIA3 mRNA.

It was shown that products of a novel CYP11A1-dependent pathway of vitamin D metabolism, such as 20(OH) D_3_ (which the present study shows to inhibit CRC proliferation) and 20,23(OH)_2_D_3_, can act as antagonists or inverse agonists on RORα and RORγ (27). These findings may not only give new insights into local (skin) or systemic regulation of genes by vitamin D metabolites, but could also explain the pleiotropic activity of 20(OH)D_3_ and 20,23(OH)_2_D_3_, without the calcemic effects (22) typically seen for classical VDR ligands (65). It should be noted that 20(OH)D_3_ has been detected in human serum (19) and epidermis (21) indicating that it may have a physiological role as an endogenous regulator.

In summary, CRC is becoming a major problem in modern societies with an aging population as its occurrence correlates with a lack of physical activity and unhealthy dietary habits, as well as with vitamin D deficiency (61,81). Thus, there is an urgent need for an inexpensive, easily available, safe and effective prevention and treatment for CRC. The present study indicates that low calcemic calcipotriol or non-calcemic 20(OH)D_3_, with proven anti-proliferative activity, may replace 1α,25(OH)_2_D_3_ in the treatment of CRC, preferably in combination with other cytostatic agents. Additionally, our results indicate that profiling the expression of genes involved in vitamin D signaling and metabolism, particularly VDR and CYP24A1, may provide a powerful tool for the planning of vitamin D_3_ analogue-based anticancer therapy.

## Figures and Tables

**Table I:** The PCR primers.

GENE	Protein name	Primer sequence (5′-3′)	Amplicon size (nt)
ACTB	β-actin	GCTCGTCGTCGACAACGGCTC	
		CAAACATGATCTGGGTCATCTTCT	353
VDR	Vitamin D receptor	CCAGTTCGTGTGAATGATGG	
		GTCGTCCATGGTGAAGGA	384
PDIA3	Protein disulfideisomerase-associated 3	CTCCGACGTGCTAGAACTCA	
		CAGGTGTTAGTGTTGGCAGT	204
CYP24A1	24-hydroxylase	GCAGCCTAGTGCAGATTT	
		ATTCACCCAGAACTGTTG	335
CYP24SV	24-hydroxylase	TCCTGAAGTTGCAGCTGGAGT	
		GAGCTCATCTATTCTGCCCATA	215
CYP2R1	25-hydroxylase	AGAGACCCAGAAGTGTTCCAT	
		GTCTTTCAGCACAGATGAGGTA	259
CYP27B1	1α-hydroxylase	TGTTTGCATTTGCTCAGA	
		CCGGGAGAGCTCATACAG	227

**Table II:** The inhibition of CRC cell proliferation by 1,25(OH)_2_D_3_ and its analogs.

	LoVo		HT29		HCT116	
			
Compound (nM)	MTT	SRB	MTT	SRB	MTT	SRB
1α,25(OH)_2_D_3_	168	0.77	57	196	47	5.9
Calcipotriol	57	0.71	ND	4.6	5.3	3.7
25(OH)D_3_	6.6	35	319	49	457	243

The data are shown as IC_50_ values (nM) representing the concentration of the secosteroid which causes a 50% decrease in cell proliferation as measured by MTT and SRB assays. ND, not determined.
